# Effects of Celecoxib and Ly117018 Combination on Human Breast Cancer Cells in Vitro

**DOI:** 10.4137/bcbcr.s2291

**Published:** 2009-04-07

**Authors:** Klaus H. Baumann, Elmar Klusmeier, Isabel Eggemann, Silke Reinartz, Achim Almeroth, Mathias Kalder, Uwe Wagner

**Affiliations:** University Hospital of Gießen and Marburg, Location Marburg, Dept. of Gynecology, Gynecological Endocrinology and Oncology, 35043 Marburg, Germany

**Keywords:** SERM, estrogen receptor, COX-2, celecoxib, Ly117018, drug synergism

## Abstract

Activation and signalling of estrogen receptor (ER) and COX-2 represent two important pathways in breast cancer cell regulation. Activation of either pathway is associated with breast cancer cell proliferation and eventually malignant progression. Raloxifene analogue, Ly117018, a selective estrogen receptor modulator and celecoxib, a specific COX-2 inhibitor have been shown to inhibit breast cancer cell proliferation when used alone in vitro and in vivo. In this study, the combined drug effects on hormone-dependent MCF-7 and hormone-independent MDA-MB-435 cells in vitro were evaluated. Cell proliferation assays excluded drug antagonism and revealed a moderate synergistic growth inhibitory activity of Ly117018 and celecoxib on both cell lines when combined in specific concentrations. Growth inhibition of either compound was not associated with cell cycle arrest. In MCF-7 cells, western blot analysis revealed a decreased phosphorylation of the AKT protein by either agent alone or in combination. In MDA-MB-435 cells, celecoxib alone induced an increase in AKT phosphorylation relative to total AKT protein; this effect was decreased in the presence of Ly117018. These results indicate that these two drugs are non-antagonistic; and when combined in specific concentrations, moderate synergistic antiproliferative activity of celecoxib and Ly117018 were observed in hormone-dependent MCF-7 and hormone-independent MDA-MB-435 cells associated with changes in cell cycle distribution and regulation of AKT protein and phosphorylation. These findings further support a central role of the ER- and COX-2 pathways in human breast cancer cells.

## Introduction

The central role of estrogen and the estrogen receptor (ER) pathway in the development and progression of hormone dependent human breast cancer has been established for decades.^1^–^5^ ER activation in human breast cancer cells leads to an increase in cell proliferation mediated at least in part by a higher transcription and bioavailability of growth factors.^2^ The modulation of intracellular signalling pathways by ER activation influences further molecular mechanisms in favour of cell survival.^6^ Reduced susceptibility to apoptosis-initiating signals contributes to uncontrolled cell growth.^7^ Up to 60% of human breast cancer have been shown to be hormone dependent.^8^,^9^ Inhibition of ER dependent tumor cell proliferation by antiestrogens represents an effective strategy of targeted therapy.^10^ Different molecular mechanisms have been identified mediating this growth inhibition by antiestrogens.^11^,^12^ However, primary or secondary antiestrogen resistance is well known in laboratory and clinical settings.^13^,^14^ Further, selective estrogen receptor modulators (SERM) have been introduced, some of which like fulvestrant, are being used in clinical practice.^15^,^16^ SERMs and estrogen depletion by aromatase inhibitors have been shown to overcome tamoxifen resistance^17^,^18^ at least in part and temporarily. Though they are already in clinical use, many aspects of how newly developed SERMs or aromatase inhibitors counteract tamoxifen resistance have not been elucidated. However, treatment resistance against these drugs has emerged as success has been established.^19^,^20^

Hence, further research is required to understand SERM mediated growth inhibition, the development of resistance to treatment, and to explore strategies to avoid or overcome this resistance. Ly117018, which is a SERM and an analogue of raloxifene has been demonstrated to inhibit ER-positive human breast cancer cell proliferation.^21^ Raloxifene reduced the incidence of hormone-dependent breast cancer in postmenopausal women.^22^

Lack of ER expression in human breast cancer cells is characterised by estrogen independent proliferation, and up to 40% of human breast cancer represent this phenotype.^8^,^9^,^23^ Adjuvant treatment strategies consist of chemotherapeutics, and of trastuzumab in case of positive HER-2 receptor status. Again, resistance to the selected treatment strategy is the major obstacle.^13^,^24^,^25^ The inhibition of cancer induced angiogenesis by antibodies or receptor tyrosine kinase inhibitors have been added to the palliative breast cancer therapy strategies.^26^–^30^

AKT represents a pivotal member in the intracellular signalling cascade. AKT is a serine-threonine kinase activated by phosphorylation.^31^,^32^ AKT mediates the activation of downstream signalling pathways leading to cell proliferation, secretion of angiogenic factors, and decreasing the susceptibility to apoptotic signals.^33^ Beside growth factors, estrogen has been shown to activate AKT by steroid receptor dependent mechanisms^34^,^35^ and by a so-called membrane initiated estrogen signalling pathway.^36^,^35^

One strategy to avoid or overcome resistance relies on the use of two or more compounds which cooperatively inhibit tumor cell proliferation. Cyclooxygenase-2 (COX-2) is one of the key enzymes for the catalytic conversion of arachidonic acid to prostanoids.^37^ Increased COX-2 expression was associated with a more malignant phenotype, partly due to an increase in the synthesisof angiogenic factors, and partly due to the suppression of immunological host responses.^38^ COX-2 expression was positively correlated with aromatase expression.^39^ Celecoxib is a COX-2 inhibitor which has been shown to inhibit proliferation of ER-positive and-negative human breast cancer cell lines.^37^

Depending on the different molecular targets, Ly117018 and celecoxib can be regarded as suitable compounds for synergistic antiproliferative activity in human breast cancer, especially in ER-positive cells; however, based on membrane mediated steroid effects, they are also useful in ER-negative breast cancer cells.

We hypothesized that the SERM Ly117018 and the COX-2 inhibitor celecoxib synergistically inhibit cell proliferation of ER-positive MCF-7 and ER-negative MDA-MB-435 human breast cancer cells in vitro. We further hypothesized, that the antiproliferative effects are associated with either modulation of AKT protein levels or phosphorylation.

## Material and Methods

### Substances

Ly117018 was provided by Eli Lilly, Bad Homburg, Ger. A stock solution of 10 mM was prepared in ethanol. Celcoxib was provided by Pfizer, Karlsruhe, Ger. A stock solution of 10 mM was prepared in DMSO.

Antibodies: Anti-AKT and anti-COX-2 were obtained from BD Bosciences (San Jose, U.S.A), anti-Aktin from Santa Cruz (Santa Cruz, U.S.A), anti-pAKT from R&D (Minneapolis, U.S.A), anti mouse and anti-rabbit from Cell Signalling New England Biolabs (Hitchin, U.K). For signal detection, ECL (Amersham, Little Chalfont, U.K) chemiluminescence detection system was used.

### Cell lines

MCF-7 and MDA-MB-435 cells were provided by R. Clarke, Lombardi Cancer Research Center, Washington, DC, U.S.A. Cells were maintained in RPMI 1640 medium containing L-glutamine, 10% heat inactivated fetal calf serum (Biokrom, Berlin, Ger; the other cell culture compounds were derived from PAA Laboratories, Pasching, Austria) and penicillin (100 U/ml) and streptomycin (100 μg/ml) at 37 °C in a humidified atmosphere with 5% CO_2_.

### Proliferation assay

Cell proliferation assays and analysis were performed and analyzed comparably as described by Leonessa et al.^40^ Cells (10^4^/ml) were distributed in 96 well plates (Greiner, Frickenhausen, Ger) and allowed to adhere for 24 hours. Then the medium was replaced by fresh medium containing vehicle or treatment reagents at the indicated concentrations. After the indicated treatment period, cells were fixed by adding 11% glutamate aldehyde (Merck, Darmstadt, Ger) solution and stained with 0.05% crystal violet (Sigma, Steinheim, Ger) in 25% methanol. After washing three times with double distilled water, cell bound crystal violet was dissolved by adding 0.1 M sodium citrate and analyzed using a photometer ELISA reader (Tecan, Grödig, Austria) at 560 nm. The number of adherent cells correlates with the crystal violet uptake and extinction.^41^ At least three independent experiments were performed.

### Calculation of drug synergism

Using the proliferation assay method described before, different concentrations of celecoxib and Ly117018 were combined. For control, wells containing vehicle only, and for calculation wells containing single treatment reagents were used. Cells were treated for 96 hours and cell content was determined as before. The interaction index was calculated as described by Tallarida.^42^ The interaction index (y) was calculated: y = a/A + b/B, where a and b represents the concentrations used in combination to achieve an antiproliferative effect, and A and B represents the concentrations of either compound alone to achieve the same effect. y < 1 indicates drug synergism; y = 1 indicates an additive drug effect. At least three independent experiments were performed.

### Cell cycle analysis

MCF-7 (6 × 10^5^/well) or MDA-MB-435 (3 × 10^5^) cells were distributed into 6-well plates (Greiner, Frickenhausen, Ger) and allowed to adhere for 24 hours. The medium was replaced by fresh medium containing vehicle or treatment reagents at the indicated concentrations. Following incubation for 96 hours, cells were harvested and fixed in 70% ethanol (Roth, Karlsruhe, Ger) at 4 °C for one hour. Following washing and treatment with RNAse (1 mg/ml stocksolution, Serva, Heidelberg, Ger) at 37 °C for 20 minutes, cells were stained with 0.01 mg/ml) propidium iodine (Calbiochem, Darmstadt, Ger) in PBS (PAA Laboratories, Pasching, Austria) containing 0.01% NaN_3_ and 2% FCS for 30 minutes at room temperature and light protected. Cell cycle distribution was determined using a FACS Calibur (BD Biosciences, San Jose, U.S.A) and analyzed using Cell Quest pro software (BD Biosciences, San Jose, U.S.A). At least three independent experiments were performed.

### Western blotting and immunodetection

Cells (1 × 10^7^) were allowed to adhere for 24 hours. The medium was replaced and cells were incubated with vehicle or indicated treatment reagent concentrations for 24 hours. Cells were harvested, washed three times in PBS and cell pellets were dissolved in lysis buffer (10 mM Tris/HCl ph 8.0, 140 mM NaCl, 3 mM MgCl_2_, 1% Triton X-100) for 30 minutes at 4 °C. Protein concentrations of cell lysates were determined using a Protein Assay Kit (Pierce, Rockford, U.S.A.) and 10 μg protein per sample were loaded on a denaturing 12.5% polyacrylamid gel. Following size separation proteins were transferred to a nitrocellulose membrane (Schleicher and Schuell, Dassel, Ger). The membranes were incubated with the indicated primary antibody and subsequently with detecting antibody; signal detection was performed using the ECL system as described by the manufacturer. X-ray films were developed, (Amersham, Little Chalfont, U.K.) scanned and analyzed by Image J software.^43^,^44^ Representative blots out of at least two independent experiments are shown.

## Results

### Inhibition of proliferation by single compounds

**Figure 1.**
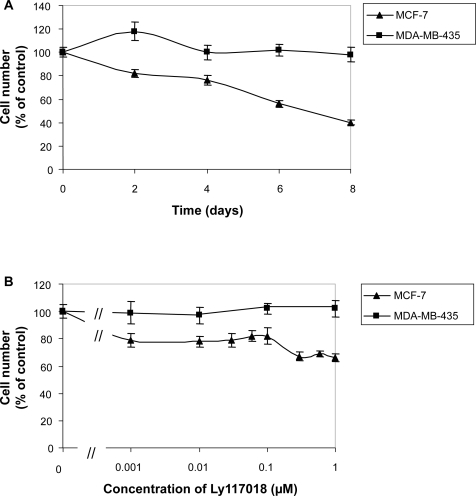
**A**) Time dependent inhibition of cell proliferation by 1 μM Ly117018. **B**) Concentration dependent inhibition of cell proliferation by Ly117018 as single agent incubated for 96 hours. Proliferation assays were performed as described in material and methods.% of control is related to vehicle treated cells. Data represent mean and standard deviation of three independent experiments. The IC50 in MCF-7 cells was 1 μM.

Time dependent growth inhibition of MCF-7 cells in vitro by Ly117018 (1 μM) was demonstrated. Incubation for up to eight days resulted in significant inhibition of proliferation (Fig. 1A) which was detectable as early as day four. The medium and reagents were replaced in case of longer incubation period after four days. Proliferation of hormone independent MDA-MB-435 breast cancer cells was not affected by Ly117018 (1 μM) during the incubation period (Fig. 1A). Inhibition of MCF-7 cell proliferation by Ly117018 was concentration dependent (Fig. 1B), whereas MDA-MB-435 cell proliferation was not influenced (Fig. 1B).

**Figure 2.**
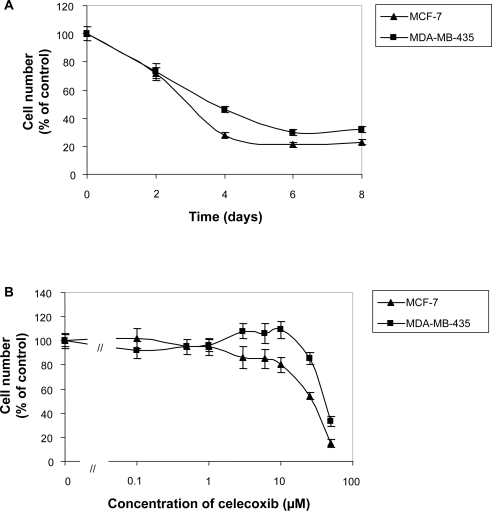
**A**) Time dependent inhibition of cell proliferation by 50 μM celecoxib. **B**) Concentration dependent inhibition of cell proliferation by celecoxib as single agent incubated for 96 hours. Proliferation assays were performed as described in material and methods.% of control is related to vehicle treated cells. Data represent mean and standard deviation of three independent experiments. The IC50 in MCF-7 cells was 27 μM, in MDA-MB-435 cells 42 μM.

Celecoxib (50 μM) was shown to inhibit proliferation of both cell lines in a time and dose dependent manner. Incubation for up to eight days with celecoxib resulted in significant inhibition of MCF-7 cell proliferation (Fig. 2A) which was detected as early as day two. The growth inhibitory effect was not caused by sole cytotoxicity, since cell number in treated cells slowly increased over time as measured by increasing crystal violet uptake. The medium and reagents were replaced in case of longer incubation period after four days. Proliferation of hormone independent MDA-MB-435 breast cancer cells was similarly affected by celecoxib during the incubation period (Fig. 2A). Inhibition of cell proliferation by celecoxib was concentration dependent (Fig. 2B) for both cell lines as shown after an incubation period of 96 hours.

### Synergism of Ly117018 and celecoxib

**Figure 3.**
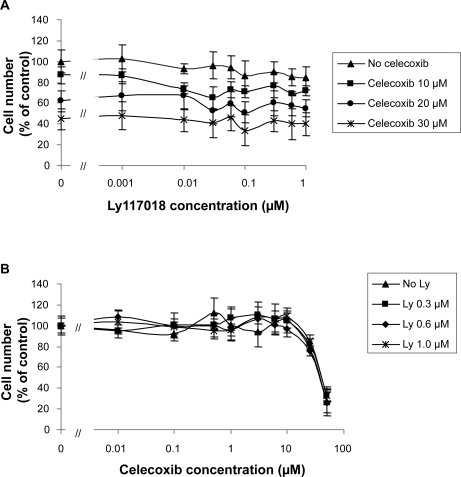
Combined effects of Ly117018 and celecoxib on MCF-7 **A**) or MDA-MB-435 **B**) human breast cancer cells. Proliferation assays were performed as described in material and methods. Cells were incubated for 96 h. These representative data show for MCF-7 cells the combination of increasing concentrations of Ly117018 ranging from 0.001 μM to 1 μM with different defined concentrations of celecoxib (10 μM, 20 μM and 30 μM). **A**) For MDA-MB435 cells, increasing concentrations of celecoxib ranging from 0.01 μM to 50 μM were combined with different defined concentrations of Ly117018 (0.3 μM, 0.6 μM and 1 μM). **B**) Data represent mean and standard deviation of three independent experiments.

Analysis of drug synergism of Ly117018 and celecoxib was performed by combining fixed concentrations of one compound with increasing concentrations of the second compound and vice versa. Cells were incubated for 96 hours and at least three independent experiments were performed for each combination. Representative data are shown in Figure 3. MCF-7 cells were incubated with different concentrations of celecoxib (10 μM, 20 μM or 30 μM) combined with increasing concentrations of Ly117018 ranging from 1 nM to 1 μM (Fig. 3A). In case of MDA-MB-435 cells, different concentrations of Ly117018 (0.3 μM, 0.6 μM or 1 μM) were incubated with increasing concentrations of celecoxib ranging from 0.1 μM to 50 μM (Fig. 3B).

The interaction index was calculated for different concentration combinations and for both cell lines (Table 1). An interaction index <1 indicates drug synergism. Ly117018 and celecoxib revealed no antagonistic activity with respect to growth inhibition of both cell lines. At distinct concentration combinations of Ly117018 and celecoxib, an interaction index value <1 was calculated (Table 1).

### Effects on cell cycle distribution

**Figure 4.**
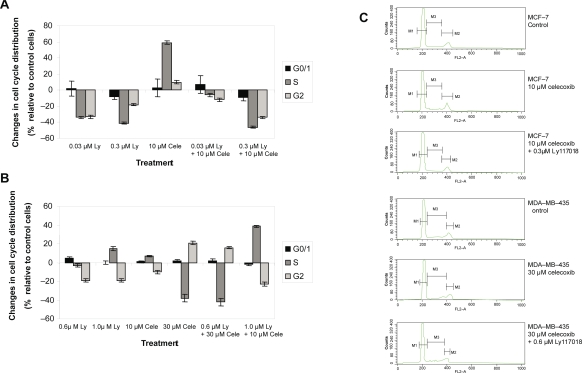
Changes in cell cycle disribution relative to vehicle treated control cells. Cells were incubated for 96 h, cell cycle analysis was performed as described in material and methods. **A**) MCF-7 cells. **B**) MDA-MB-435 cells. Data show mean and standard deviation of three independent experiments. **C**) Representative flow cytometry graphs. The statistical significance level of p ≤ 0.05 was not reached (nonparametric Mann-Withney-Wilcoxon Test).

After an incubation period of 96 hours, Ly117018 (0.03 or 0.3 μM) and 10 μM celecoxib revealed a mixed response pattern in MCF-7 cells. Ly117018 as a single agent reduced number of cells in G2 and S phase of the cell cycle; celecoxib used as a monocompound resulted in an increase in the number of cells in G2 and S-phase. The combination of both resulted in a decrease in the number of cells in G2 and S-phase (Fig. 4).

In MDA-MB-435 cells, Ly117018 (0.6 or 1.0 μM) resulted in a decrease of cell number in G2-phase while celecoxib, (30 μM) when used alone induced a decrease of cell number in the S-phase accompanied by an increase in G2. Given together Ly117018 (0.6 μM) and celecoxib (30 μM) induced changes in cell cycle distribution comparable to the effects induced by celecoxib alone. In contrast, 1.0 μM Ly117018 combined with 10 μM celecoxib resulted in a cell cycle distribution pattern similar to Ly117018 alone (Fig. 4).

### Effects on AKT protein

**Figure 5.**
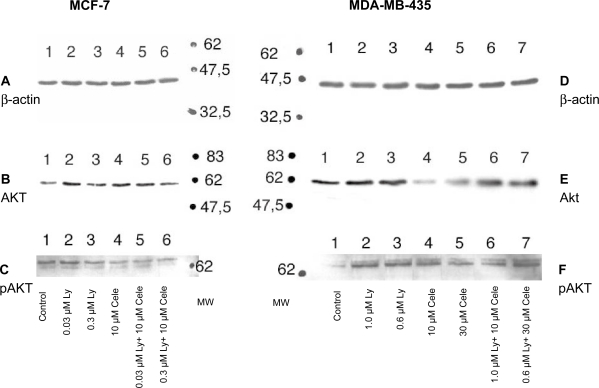
Western blots were performed and analyzed as described in material and methods. The figure shows for MCF-7 (A, B, C) and MDA-MB-435 cells (D, E, F) one representative experiment out of at least two independent experiments. Cells were treated for 24 h. **Lanes in A, B, C:** MCF-7 cells treated with vehicle (1), 0.03 μM Ly117018 (2), 0.3 μM Ly117018 (3), 10 μM celecoxib (4), 0.03 μM Ly117018 + 10 μM celecoxib (5), 0.3 μM Ly117018 + 10 μM celecoxib (6). **Lanes in D, E, F:** MDA-MB-435 cells treated with vehicle (1), 1.0 μM Ly117018 (2), 0.6 μM Ly117018 (3), 10 μM celecoxib (4), 30 μM celecoxib (5), 1.0 μM Ly117018 + 10 μM celecoxib (6), 0.6 μM Ly117018 + 30 μM celecoxib (7). MW = molecular weight marker. For beta-Actin, AKT and pAKT, separate blots were stained.

The relative amounts of AKT protein and AKT phophorylation were investigated by western blotting and immunodetection. MCF-7 and MDA-MB-435 cells were incubated for 24 hours with the indicated drug concentrations and combination. In Figure 5, the results of one experiment is shown which is representative of two independent experiments. Detection of beta-Actin served as loading and transfer control. In MCF-7 cells, Ly117018 and celecoxib treatment were associated with an increase of AKT protein relative to vehicle treated control. In MDA-MB-435 cells, Ly117018 treatment resulted in a small increase of AKT protein, whereas celecoxib as a single agent led to a marked decrease of AKT protein. Ly117018 and celecoxib in combination resulted in an increase of AKT protein in MDA-MB-435 cells compared to celecoxib alone.

**Figure 6.**
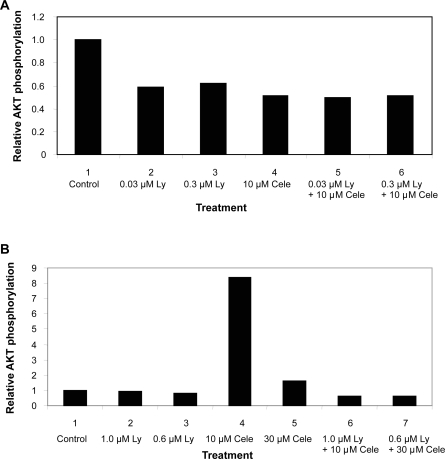
Semiquatitative analysis of western blots. Data show the relative amount of phosphorylated AKT to total AKT. **A**) MCF-7 cells, calculated from figure 5. Cells treated with vehicle (1), 0.03 μM Ly117018 (2), 0.3 μM Ly117018 (3), 10 μM celecoxib (4), 0.03 μM Ly117018 + 10 μM celecoxib (5), 0.3 μM Ly117018 + 10 μM celecoxib (6). **B**) MDA-MB-435 cells treated with vehicle (1), 1.0 μM Ly117018 (2), 0.6 μM Ly117018 (3), 10 μM celecoxib (4), 30 μM celecoxib (5), 1.0 μM Ly117018 + 10 μM celecoxib (6), 0.6 μM Ly117018 + 30 μM celecoxib (7), data calculated from Figure 5.

Evaluation of pAKT demonstrated a decrease of pAKT protein level in MCF-7 cells induced by Ly117018, celecoxib or both. Even more, the relative amount of phophorylated AKT to total AKT decreased upon treatment with Ly117018, celecoxib or both (Fig. 6). Evaluation of phosphorylated AKT in MDA-MB-435 cells showed a marked increase in the relative amount of phosphorylated AKT to total AKT due to celecoxib treatment alone, which was not detectable in the presence of Ly117018 (Fig. 6).

## Discussion

The presented data describe for the first time, the combined antiproliferative effects of the SERM Ly117018 and the COX-2 inhibitor celecoxib on human breast cancer cells in vitro. Using ER-positive MCF-7 cells, the results not only exclude an antagonism of both compounds but also reveal a moderate synergism when combined in specific concentrations. The influencing effects of Ly117018 and celecoxib on MDA-MB-435 cells further point to the notion that SERMs like Ly117018 exert biological effects independent of ER expression. The results achieved in both cell lines support the hypothesis of the interaction of different signalling pathways in human breast cancer.

Ly117018 and raloxifen have been demonstrated to exert antiproliferative effects on human breast cancer cell lines in vitro and in vivo.^21^,^45^ IC50 values were calculated to determine the antiproliferative potency of different SERMs.^46^ In addition to cell cycle perturbation and arrest, the induction of apoptosis is a mechanism further associated with SERM induced growth inhibition.^47^,^48^

With respect to ER-negative, hormone-independent breast cancer cell lines, SERMs have been demonstrated to exert some antiproliferative activity in vitro^49^,^50^ by mechanisms which have not been elucidated, although other findings have been published.^51^

Celecoxib is a COX-2 inhibitor which has been demonstrated to exert antiproliferative effects on ER-positive and-negative human breast cancer cell lines in vitro and in vivo.^37^ Those published observations are similar to these findings demonstrating a time and dose dependence of cell growth inhibition of MCF-7 and MDA-MB-435 cells.

SERMs have been shown to influence the arachidonic acid pathway thus influencing the biological role of COX-2. COX-2 influences aromatase expression and downstream molecular signalling pathways cross ER-induced cascades.^39^,^52^–^54^ Eventually, SERMs might be able to influence molecular pathways by membrane mediated steroid effects.^35^ These observations support the necessity to investigate the interaction of SERMs and COX-2 inhibitors, mainly to exclude antagonizing effects and also for evaluating synergistic activities.

Elevated COX-2 protein levels were demonstrated in human breast cancer tissues.^55^,^56^ Increased COX-2 expression was shown to be associated with a more malignant phenotype^57^ including the over expression of the HER-2 receptor activity.^58^ In contrast, in ER positive breast cancer, elevated COX-2 levels are not associated with poor outcome.^59^ Additionally, normal epithelial breast tissue surrounding a ductal carcinoma in situ (DCIS) contained higher COX-2 levels than the DCIS epithelium.^60^ In a larger series of normal breast tissue, DCIS, and invasive breast cancers, COX-2 protein and mRNA levels were lower in invasive breast cancer tissues and thus does not support the hypothesis correlating increased COX-2 levels with malignant progression.^61^ Thus, COX-2 protein levels and expression might not be an appropriate parameter to determine the relevance of COX-2 in tumor progression.

COX-2 activation promotes lymphatic invasion of breast cancer cells.^62^ This was confirmed by transfection of human breast cancer cells with a COX-2 expression vector leading to higher COX-2 protein levels and a more aggressive phenotype than parental cells.^63^ COX-2 and prostaglandin E2 induced the expression of Her-2 receptors characterizing a more malignant phenotype.^64^ Inhibition of COX-2 resulted in a decreased Her-2 receptor expression and also sensitized cells for the anti proliferative effects of trastuzumab.^65^ This sensitizing potential of COX-2 inhibitors was further confirmed with respect to doxorubicin treatment, where increased anti proliferative effects of doxorubicin due to celecoxib were mediated in part by NFkB.^66^

Increased COX-2 activity resulted in resistance to tamoxifen.^52^ Elevated COX-2 activity was not associated with reduced doxorubicin or taxane sensitivity.^52^ The aforementioned observations point to the important role of the COX-2 signalling pathway and the eicosanoid system in hormone-dependent and-independent breast cancer cells. The relevance is well beyond the angiogenic potential of COX-2 but also based on the capability to interact with estrogen receptor and growth factor receptor activated signalling pathways.^67^

A more recent comprehensive investigation has provided strong evidence that the role of COX-2 is far from being fully elucidated. Stably transfected COX-2 knockdown Hs578T and 21MT-1 cells did not change with respect to their proliferative or invasive capacity.^61^

AKT has a central role in intracellular signalling. In MCF-7, cells the inhibition of cell proliferation by Ly117018 and celecoxib was associated with a decrease of phosphorylated AKT. In ER-negative MDA-MB-435 cells, treatment with celecoxib led to a decrease of total AKT protein. The decrease of total AKT protein was less in the presence of both compounds. The decrease of phorshorylated AKT observed in MCF-7 cells was not present in MDA-MB-435 cells. Celecoxib increased the relative amount of phosphorylated AKT protein in MDA-MB-435 cells; but this effect was reduced upon cotreatment with Ly117018. The effect of Ly117018 in MDA-MB-435 cells might be caused in part by membrane initiated SERM signalling. In vitro investigations using different human breast cancer cell lines demonstrated the reduction of AKT activation by celecoxib or Ly117018 treatment.^68^–^70^ Increased activation of AKT was shown to result in an increased estrogen receptor alpha activation, which was also attributed for causing resistance to tamoxifen.^71^ Constitutively, increased AKT activation resulted in an upregulation of COX-2 protein.^72^ Thus, the interaction of the ER- and the COX 2-signalling pathways are of further interest.

Celecoxib treatment resulted not only in G0/G1 arrest and reduction of cells in S-phase, but also in the induction of apoptosis.^37^,^73^ Our data revealed no G0/G1 arrest in both cell lines due to celecoxib treatment. The S-phase proportion is not uniformly altered as is obvious from Figure 4. Perturbations in cell cycle distribution like G2/M phase accumulation preceding growth inhibition and apoptosis were observed in a variety of breast cancer cell lines upon treatment with different compounds.^74^,^75^

Targeting the estrogen receptor with SERMs is an effective treatment in early and metastasized breast cancer, although the problem of SERM resistance is an unresolved issue.^18^ The relevance of COX-2 expression and targeting by COX-2 inhibitors like celecoxib is an exciting experimental and clinical research area. Early clinical data indicate a chemopreventive and a chemosensitizing activity of COX-2 inhibitors in breast cancer.^76^,^77^ Already initiated or planned clinical trials will further elucidate the significance of COX-2 inhibition in prevention and treatment of breast cancer.^77^–^79^

## Conclusion

The experimental in vitro data presented herein strengthen the notion of the antiproliferative potency of the COX-2 inhibitor, celecoxib on hormone-dependent and -independent breast cancer cells in vitro, confirm the growth inhibitory activity of the SERM Ly117018 in ER-positive breast cancer cells, support the hypothesis of membrane initiated SERM signalling effects, and above all exclude drug antagonism of Ly17018 and celecoxib in the investigated ER-positive and- negative human breast cancer cells in vitro.

## Figures and Tables

**Table 1.** Interaction index, calculated for an incubation period of four days. Single agent concentrations were calculated from the experiments showing dose dependent effects (Fig. 2) achieving the same proliferation inhibition as combined agents with listed concentrations.

**MCF-7**		
**Concentration of celecoxib [μM]**	**Concentration of Ly117018 [μM]**	**Interaction index**
10	0.03	0.039
30	0.1	0.76
20	0.03	0.8
	**MDA-MB-435**	
30	0.6	0.76
40	0.3	0.88
25	0.3	0.78

## Abbreviations

ER: estrogen receptor;
SERM: selective estrogen receptor modulator;
pAKT: phosphorylated AKT protein;
IC: inhibitory concentration.
